# The Mitochondrial Fission Adaptors Caf4 and Mdv1 Are Not Functionally Equivalent

**DOI:** 10.1371/journal.pone.0053523

**Published:** 2012-12-31

**Authors:** Qian Guo, Sajjan Koirala, Edward M. Perkins, J. Michael McCaffery, Janet M. Shaw

**Affiliations:** 1 Department of Biochemistry, University of Utah School of Medicine, Salt Lake City, Utah, United States of America; 2 Integrated Imaging Center, Department of Biology, Johns Hopkins University, Baltimore, Maryland, United States of America; Institute of Biology Valrose, France

## Abstract

Mitochondrial fission in eukaryotes is mediated by protein complexes that encircle and divide mitochondrial tubules. In budding yeast, fission requires the membrane-anchored protein Fis1 and the dynamin-related GTPase Dnm1. Dnm1 is recruited to mitochondria via interactions with the adaptor proteins Caf4 and Mdv1, which bind directly to Fis1. Unlike Mdv1, a function for Caf4 in mitochondrial membrane scission has not been established. In this study, we demonstrate that Caf4 is a bona fide fission adaptor that assembles at sites of mitochondrial division. We also show that fission complexes may contain Caf4 alone or both Caf4 and Mdv1 without compromising fission function. Although there is a correspondence between Caf4 and Mdv1 expression levels and their contribution to fission, the two adaptor proteins are not equivalent. Rather, our functional and phylogenetic analyses indicate that Caf4 mitochondrial fission activity has diverged from that of Mdv1.

## Introduction

An ancient genome wide duplication in the lineage including the budding yeast *Saccharomyces cerevisiae* played an important role in the evolution of this singled celled eukaryote [1], [2]. After this event, duplicated genes (paralogs) experienced accelerated evolution, most often involving one member of the gene pair. Of the 5885 existing *S. cerevisiae* genes, 450 are paralog pairs [3], [4]. For many of these, it remains unclear whether one of the genes has acquired a new function, maintains a redundant function, or has developed a specialized function within the same cellular process.

Mitochondrial fission is one cellular process that utilizes paralogous genes. Yeast mitochondrial membranes form tubular structures that undergo frequent fission and fusion events [5], [6]. When fission is blocked, ongoing fusion creates a single, interconnected mitochondrial net [7]–[9]. A previous study demonstrated that defects in mitochondrial fission have a negative fitness cost in yeast, since these abnormal nets are inefficiently partitioned into newly formed daughter cells during meiotic division, and the resulting spores are inviable [10]. Successful mitochondrial fission requires an adaptor protein called Mdv1 [11], [12], which bridges the interaction between the cytoplasmic dynamin-related GTPase Dnm1 and the mitochondrial outer membrane-anchored protein Fis1 [13]–[15]. On the membrane, Mdv1 promotes the assembly of Dnm1 into spirals that often surround the mitochondrial tubule [16], [17]. A subset of these structures are located at sites on mitochondria that ultimately divide [17], [18].

**Figure 1:**
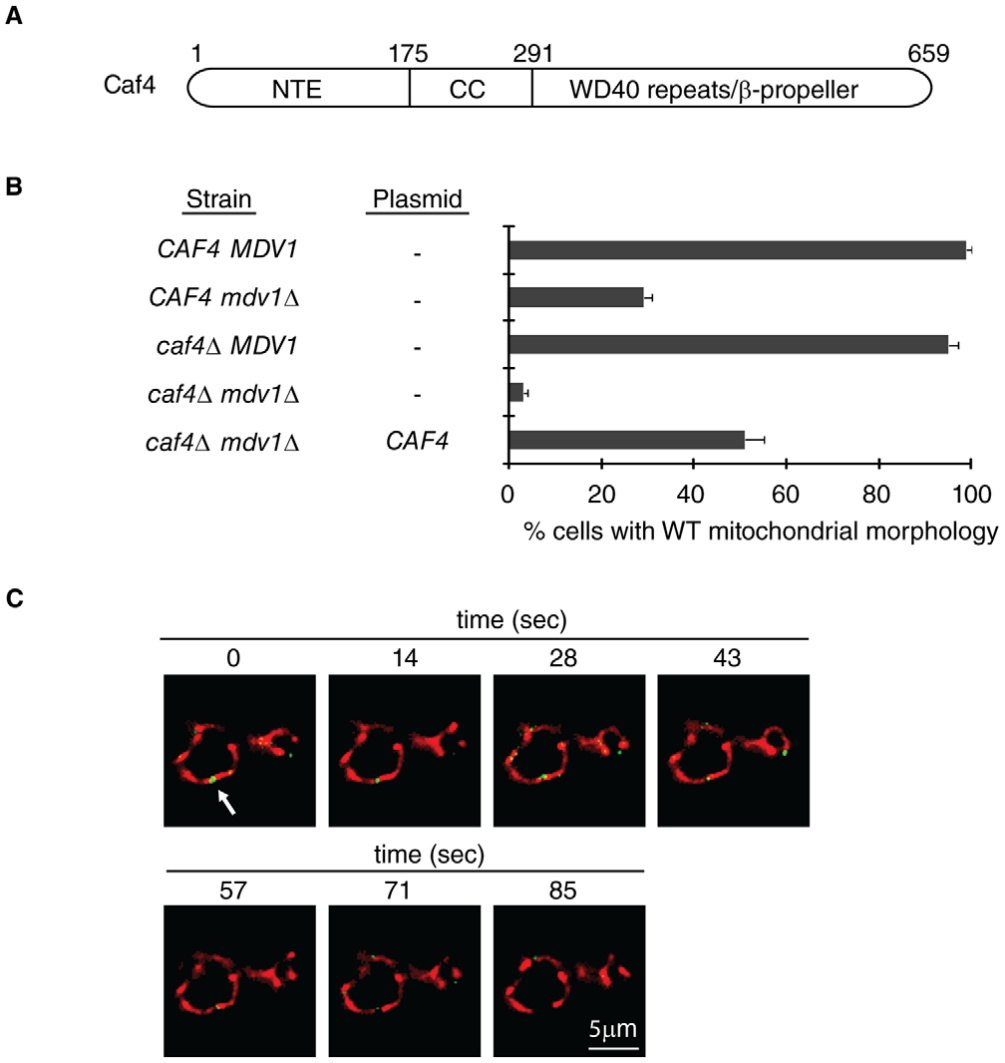
Caf4 functions independently as a mitochondrial fission adaptor. (**A**) Domain structure of the Caf4 fission adaptor, including the N-terminal extension (NTE), predicted coiled-coil (CC), and WD40 repeats predicted to form a β-propeller (WD40 repeats/ β-propeller). (**B**) Quantification of mitochondrial morphology in the indicated strains (n = 100 cells). Bars and error bars are the mean and SD of three independent experiments. (**C**) Time-lapse imaging of a mitochondrial fission event mediated by GFP-Caf4 expressed in a *caf4Δ mdv1Δ* strain. Mitochondria are labeled with mt-RFP. Scale bar: 5 µm.

An Mdv1 paralog called Caf4 was identified in a directed proteomics screen [19]. Like Mdv1, the Caf4 adaptor is composed of three domains, an N-terminal extension (NTE), a coiled-coil (CC) and C-terminal WD40 repeats predicted to form a β-propeller (Figure 1A). The NTE domains of Mdv1 or Caf4 form a clamp around the Fis1 TPR-like domain, which mediates adaptor localization to the mitochondrial surface [20]. The Mdv1 and Caf4 WD40/β-propeller domains interact with, and recruit, Dnm1 to the membrane [14], [15], [19]. Structural studies reveal that Mdv1 dimerizes via an antiparallel coiled-coil [21], [22], and Caf4 is thought to dimerize by a similar mechanism. Caf4 and Mdv1 also interact in vivo [19], though whether this occurs via coiled-coil formation between the two proteins is unclear.

Yeast cells lacking Mdv1 exhibit severe mitochondrial morphology defects, establishing an essential role for this adaptor in fission [11], [12]. By contrast, loss of Caf4 function enhances mitochondrial fission defects when Mdv1 is absent, but does not cause obvious fission defects on its own [19]. Although Caf4 can function to recruit Dnm1 to mitochondria in vivo, it has not been shown to directly participate in membrane scission. Moreover, it is not clear whether there is functional divergence between the two adaptor proteins.

In this study, we directly test the function of Caf4 in mitochondrial fission. We show that Caf4 is a bona fide fission adaptor that assembles at sites of mitochondrial division. Although Caf4 can function alone as an adaptor, complexes containing both Caf4 and Mdv1 exhibit no obvious defects in mitochondrial fission. Genomic swapping studies indicate that Caf4 cannot substitute for Mdv1 in vivo. Moreover, over expression of Caf4 (but not Mdv1) from a regulated promoter induces dominant negative fission defects. When combined with phylogenetic analysis, these findings establish that Caf4 mitochondrial fission activity has diverged from that of Mdv1.

## Results

### Caf4 is present at mitochondrial fission sites and is sufficient for fission

A genome-wide analysis of yeast protein expression showed that the abundance of Caf4 is one sixth that of Mdv1 [23]. This finding raised the possibility that lower expression of Caf4 relative to Mdv1 was responsible for the functional differences observed for the two fission adaptors. We tested this idea by examining the effect of increasing Caf4 expression on mitochondrial fission in vivo. As shown in Figure 1B, 100% of wildtype (WT, *CAF4 MDV1*) cells expressing Caf4 and Mdv1 had branched, tubular mitochondria, while cells lacking both adaptors had severe fission defects (*caf4*Δ *mdv1*Δ, 3% cells with WT morphology). Similar to previous reports [19], an *mdv1*Δ strain expressing endogenous Caf4 exhibited significant fission defects (*CAF4 mdv1*Δ, 29% cells with WT morphology), and a *caf4*Δ strain expressing endogenous Mdv1 exhibited essentially no fission defects (*caf4*Δ* MDV1*, 95% cells with WT morphology). Importantly, when Caf4 expression was induced from the *MET25* promoter on a plasmid in the *caf4*Δ * mdv1*Δ strain, the population of cells with WT mitochondrial morphology increased to 51%. This increased rescue of fission defects was correlated with a 5.4 fold increase over native Caf4 expression (data not shown).

Both decreased fusion and increased fission could generate WT mitochondrial morphology in the previous experiment. Time-lapse imaging studies confirmed that Caf4 in these cells was mediating mitochondrial fission when Mdv1 was absent. By epifluorescence microscopy, Dnm1-containing fission complexes appear as puncta that colocalize with mitochondrial tubules [8]. Mdv1 or Caf4 also appear in these puncta after coassembly with Dnm1 [11], [12], [19]. In *mdv1*Δ *caf4*Δ cells expressing GFP-Caf4 protein, green Caf4 puncta were observed on mt-RFP labeled mitochondrial tubules at sites where fission occurred (Figure 1C arrow, a representative example is shown). Together, these results indicate that Caf4 can function independently as a fission adaptor in yeast.

### The Caf4 and Mdv1 adaptors are not functionally equivalent

**Figure 2:**
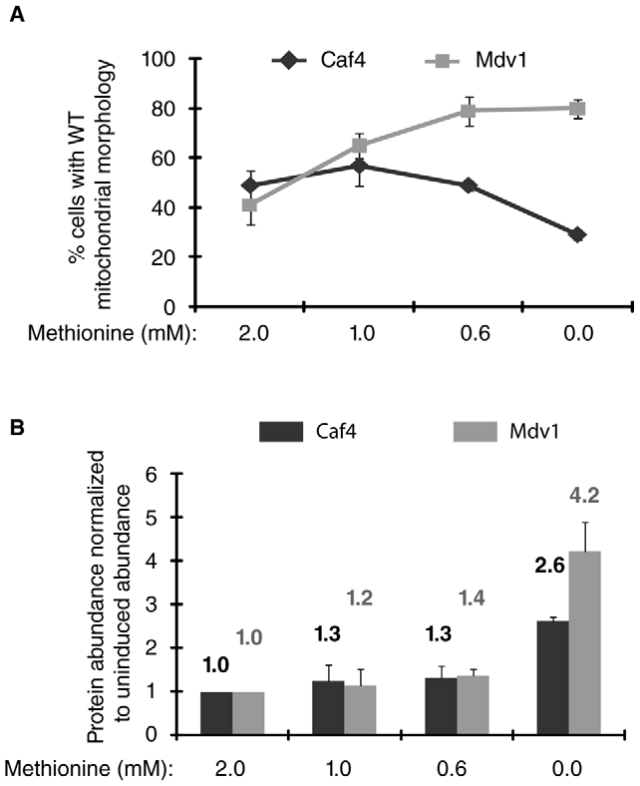
Caf4 and Mdv1 are not functionally equivalent. (**A**) Quantification of mitochondrial morphology in a *caf4*Δ *mdv1*Δ strain expressing Caf4 or Mdv1 from the repressible *MET25* promoter in media containing different methionine concentrations (n = 100 cells). (**B**) Protein abundance of Caf4 or Mdv1 expressed from the repressible *MET25* promoter in difference methionine concentrations. The abundance of each protein was subsequently normalized to its abundance in medium containing 2.0 mM methionine. Bars and error bars are the mean and SD of three independent experiments.

Although Caf4 can carry out fission in the absence of Mdv1, the two adaptors may not be functionally equivalent. To test this possibility, Caf4 and Mdv1 were expressed from the *MET25* promoter in a *caf4*Δ *mdv1*Δ strain, and the abundance of each adaptor was increased by reducing the methionine concentration in the medium. WT mitochondrial morphology increased from 41% to 80% (Figure 2A) when Mdv1 steady state abundance was raised 4.2 fold by removing methionine from the medium (Figure 2B). By contrast, raising Caf4 steady-state abundance 1.3 fold (1.0 mM methionine, Figure 2B) initially increased WT mitochondrial morphology from 49% to 57% (Figure 2A). Further induction to 2.6 fold Caf4 over expression (0.0 mM methionine) reduced the rescue to 29%, indicating that increasing Caf4 expression had a dominant negative effect on mitochondrial fission. Although we did not observe dominant-negative fission defects by increasing Mdv1 expression 4.2 fold (0.0 mM methionine) in these studies, we were able to induce dominant-negative defects by over expressing Mdv1 from the inducible *GAL1* promoter (∼20 fold induction, data not shown) [14]. The dominant-negative effect caused by Caf4 overexpression is likely due to Caf4′s ability to interact with and sequester both Fis1 and Dnm1. In addition, differences in Caf4 sequence and structure may contribute to its ability to disrupt fission when overexpressed. These different effects of Caf4 and Mdv1 over expression on mitochondrial fission support the idea that the fission adaptor functions of the two proteins are not equivalent.

**Figure 3:**
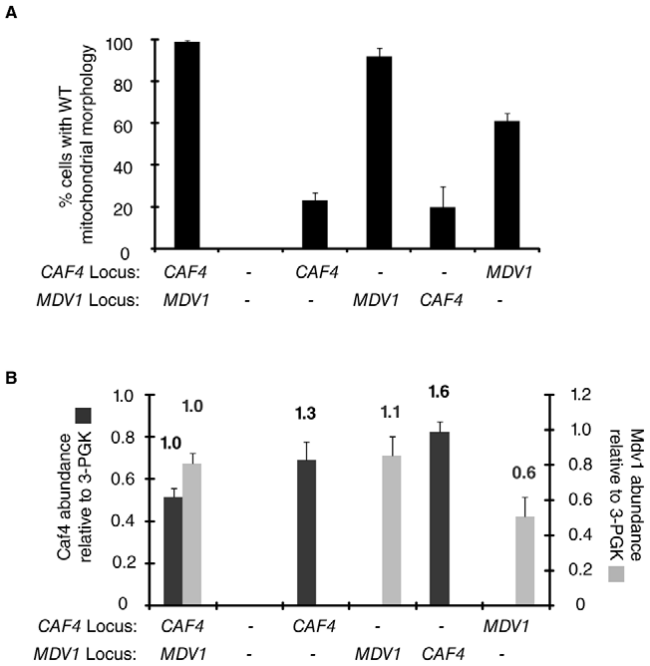
Caf4 causes dominant-negative fission defects when expressed from the *MDV1* promoter at the *MDV1* locus. (**A**) Quantification of mitochondrial morphology in the indicated strains (n = 100 cells). (**B**) Steady-state abundance of Caf4 and Mdv1 in strains shown in (**A**). Owing to the use of different antibodies, the abundance of Caf4 and Mdv1 in the same and different strains should not be compared to one another. Bars and error bars are the mean and SD of three independent experiments.

We also tested whether expression of Caf4 from the native *MDV1* promoter and locus was sufficient to replace Mdv1 function. Mitochondrial morphology was WT in cells expressing only Mdv1 from its native locus (Figure 3A). When Caf4 alone was expressed from the *MDV1* promoter, Caf4 abundance increased 1.6 fold over the endogenous level (Figure 3B) but mitochondrial fission remained defective (Figure 3A, 20% WT morphology). (Note that this 1.6 fold increase is lower than the level of Caf4 expression shown to cause dominant negative fission defects in Figure 2). Thus, Caf4 expressed from the *MDV1* locus is not able to support WT levels of mitochondrial fission. By contrast, we observed a 0.6 fold decrease in expression of Mdv1 from the *CAF4* promoter with a corresponding decrease in WT mitochondrial morphology. We also noted a reproducible 1.3 fold increase in Caf4 expression from its native locus when the *MDV1* coding region was deleted. However, Mdv1 abundance did not change significantly when *CAF4* was absent. Thus, regulatory circuits that control expression from the *CAF4* gene may be able to sense and respond to changes in cellular Mdv1 abundance.

### Caf4 and Mdv1 can work together at a mitochondrial fission site

Mdv1 self-assembles via dimerization of an internal coiled-coil [21], [22] and, based on structural predictions and two hybrid assays, Caf4 is predicted to behave in a similar manner. Caf4 and Mdv1 can also interact with one another [19]. However, it is not known whether Caf4 and Mdv1 can function together during mitochondrial fission. To address this question, we examined the extent and function of Caf4 and Mdv1 colocalization in vivo.

**Figure 4:**
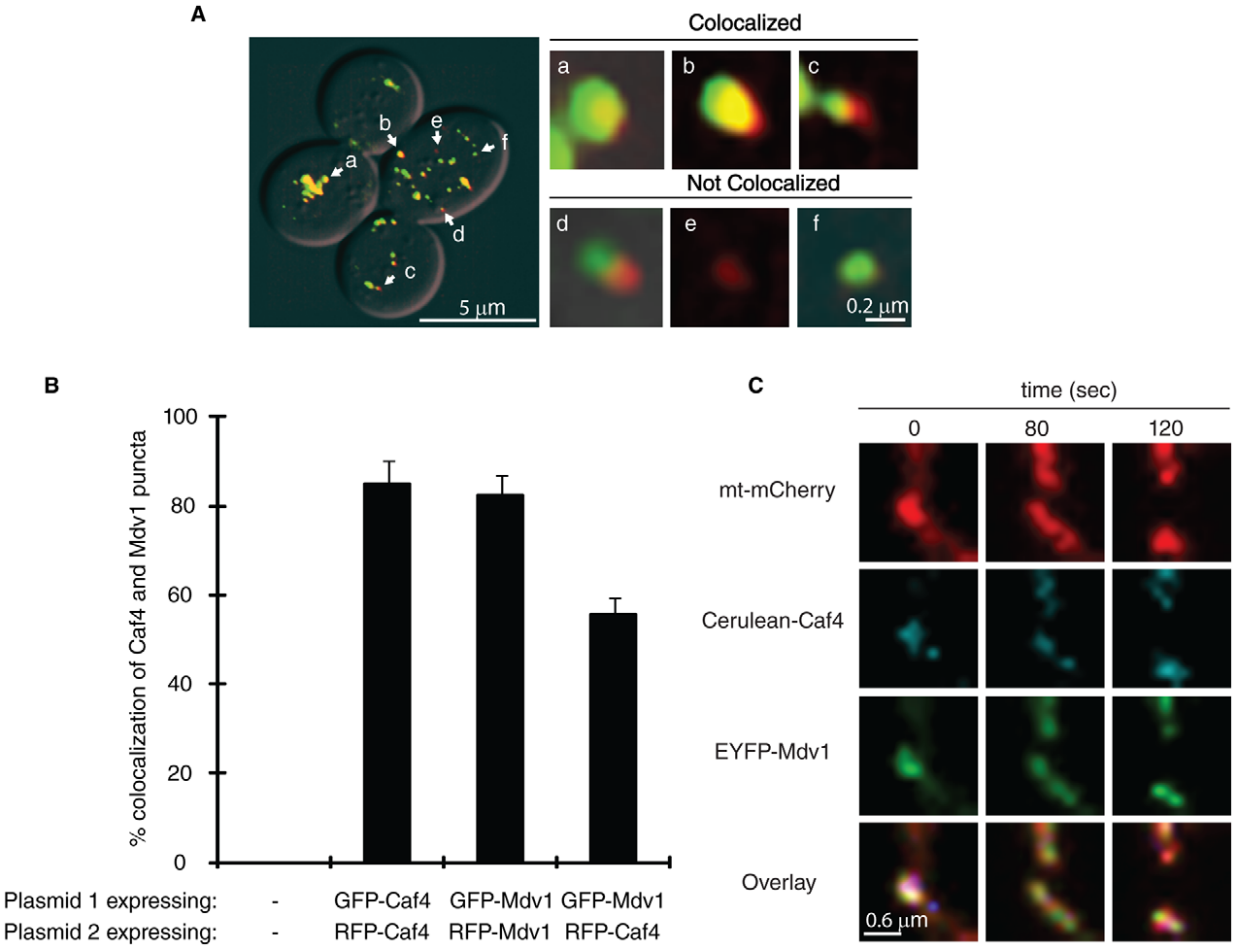
Mitochondrial puncta containing both Caf4 and Mdv1 are fission competent. (**A**) Large panel at the left shows a representative image of *caf4*Δ *mdv1*Δ cells expressing GFP-Caf4 and RFP-Mdv1. Scale bar: 5 μm. a-f marks puncta enlarged from the image in (A) at the left. Examples of partially or completely co-localized (a–c) versus isolated (d–f) puncta are shown. Scale bar: 0.2 μm. (**B**) Quantification of Caf4 and Mdv1 colocalization. GFP- or RFP-tagged Caf4 and Mdv1 were expressed in *caf4Δ mdv1Δ* cells in the pair-wise combinations indicated. The number of GFP puncta colocalized with RFP puncta was quantified and normalized to the total number of RFP puncta in each cell (n = 10 cells). Bars and error bars are the mean and SD of three independent experiments. (**C**) Time lapse imaging of a mitochondrial fission event at a site where Caf4 and Mdv1 co-localize. Cerulean-tagged Caf4 (shown in cyan pseudo-color) and EYFP-Mdv1 were integrated at the *HO* and *MDV1* loci, respectively. Mitochondria were visualized using plasmid-borne mt-mCherry. The overlay contains true color images of all three channels. White areas indicate regions where signals from all three fluorescent proteins overlap. Scale Bar: 0.6 μm.

We began by quantifying the colocalization of GFP-Caf4 with RFP-Caf4 and GFP-Mdv1 with RFP-Mdv1 in cells lacking WT versions of the two proteins. Since fluorescent puncta in these experiments contain a single fission adaptor coassembled with Dnm1, these results represent the maximum colocalization we would expect to observe. Representative images of the categories scored are shown in Figure 4A (colocalized, a–c; not colocalized, d–f). Using this approach, 85% of Caf4 and 82% of Mdv1 signals colocalized in these studies, with the majority of puncta displaying the colocalization pattern shown in Figure 4A, a and b (72% and 81% respectively).

Next we quantified the colocalization of GFP-Caf4 and RFP-Mdv1 in mitochondrial puncta. We observed that 56% of RFP-Caf4 puncta colocalized with GFP-Mdv1 puncta (Figure 4B). Of these 56%, the majority (77%) displayed the colocalization pattern shown in Figure 4A, a and b. Similar results were obtained with GFP-Caf4 and RFP-Mdv1 (data not shown), indicating that placement of the fluorescent protein tags did not affect the experimental results.

Further analysis established that mitochondrial fission occurred at sites where Caf4 and Mdv1 colocalized. As shown in Figure 4C, time-lapse imaging studies of cells expressing Cerulean-Caf4 and EYFP-Mdv1 showed that puncta containing both adaptors (t = 0 s, white signal in overlay) were able to mediate fission of an mCherry-labeled mitochondrial tubule. In the example shown, fission was complete after 80 seconds, at which point Caf4 and Mdv1 disassembled and dispersed along the surface of the separated tubules. These data provide a direct demonstration that Caf4 and Mdv1 can work together at the same mitochondrial fission site in vivo.

### Evolution of the fungal fission adaptors

**Figure 5:**
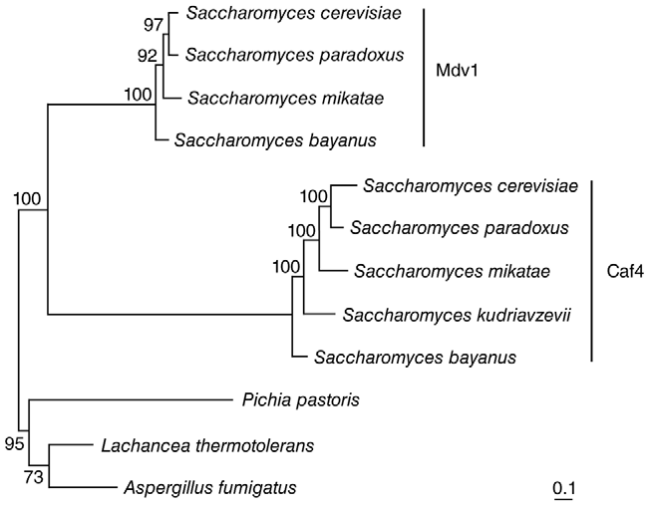
Phylogenetic relationship of Caf4 and Mdv1 in representative fungi. The amino acid sequences of paralogs from fully sequenced fungal genomes were aligned using ClustalW2 and a phylogenetic tree was constructed using the maximum-likelihood method. Bootstrap values above 50 are shown at the nodes of the branches. Branch lengths are proportional to the number of amino acid substitutions per site. The Caf4 and Mdv1 clades are marked by vertical lines. Scale bar: 0.1 substitution per site in the protein.

A major event in the evolutionary history of *Saccharomyces cerevisiae* was an ancestral whole genome duplication, which was followed by elimination of many redundant gene copies during the divergence of species in the genus *Saccharomyces* [1], [2]. Our phylogenetic analysis indicates that *CAF4* and *MDV1* are among those redundant gene copies that have been retained during evolution. As shown in Figure 5, *Pichia pastoris*, *Lachancea thermotolerans* and *Aspergillus fumigatus* contain only a single gene that is homologous to *CAF4/MDV1*. Duplication of this ancestral gene gave rise to *CAF4* and *MDV1* in most *Saccharomyces* species, consistent with an origin resulting from whole genome duplication. The increased branch lengths in the Caf4 clade compared to the Mdv1 clade indicate that the Caf4 protein has undergone more amino substitutions than Mdv1 since the original duplication event. This observation suggests that Mdv1 is under tighter constraints to perform its essential function in mitochondrial fission, consistent with our functional analysis. One possible outcome of this accelerated rate of Caf4 protein evolution is that the *CAF4* gene will ultimately be lost from the *Saccharomyces* genome. However, it appears that *MDV1*, but not *CAF4*, was lost from *Saccharomyces kudriavzevii*. Thus, Caf4 can either substitute for Mdv1 function or a completely different protein has assumed the essential role in membrane fission in this species. An alternative outcome in species with both proteins is that Caf4 will acquire additional or novel function(s) (see discussion).

## Discussion

Prior to this study, the Caf4 adaptor was known to function early in fission to recruit Dnm1 to the outer mitochondrial membrane. However, whether Caf4 participated in mitochondrial membrane fission after Dnm1 recruitment was unclear. Our results provide a direct demonstration that, in the absence or presence of Mdv1, Caf4 localizes in complexes on mitochondria that carry out membrane division.

It has been suggested that Caf4 serves as regulator of mitochondrial fission. We think it is unlikely that Caf4 acts as a positive regulator, since its presence or absence has little effect on Mdv1-mediated fission in WT cells. Several observations also suggest that Caf4 does not act as a negative regulator. When Caf4 is expressed in the absence of Mdv1, fission occurs and the time course of fission is similar to WT. In addition, when Caf4 and Mdv1 are both present in mitochondrial puncta, fission appears to occur normally. However, Caf4 and Mdv1 are not functionally equivalent. When expressed at maximum levels from the *MET25* promoter, Caf4 (but not Mdv1) causes dominant-negative fission defects. Moreover, expression of the *CAF4* gene from the *MDV1* promoter in the genome is not sufficient to restore mitochondrial fission to levels observed in an *MDV1 caf4*Δ strain.

In many instances, one member of a duplicated gene pair is retained during evolution because it develops a specialized function (subfunctionalization) within a cellular process or because it acquires an entirely new function (neofunctionalization) [24]. There is evidence that Caf4 has acquired a specialized function in mitochondrial biology. Jakob and colleagues showed previously that Caf4 (but not Mdv1) plays a role in orienting a subset of mitochondrial Dnm1 puncta toward the yeast cell cortex [18]. This Dnm1 orientation is proposed to anchor mitochondria near the plasma membrane and distribute them at the cell periphery. Caf4 has also been shown to participate with Dnm1, Mdv1 and Fis1 in peroxisome division [25], [26]. Although it is possible that Caf4 has acquired specialized functions in peroxisome division, we do not observe significant changes in peroxisome number or morphology in *caf4*Δ cells relative to WT (Figure S1B and C). Moreover, cells lacking Caf4 grow as well as WT on carbon sources that require peroxisome function (i.e. oleate, Figure S1A). If Caf4 had acquired a new function in a critical cellular process, loss of Caf4 would be expected to affect yeast fitness. However, our analysis of *caf4*Δ cells has not uncovered conditions that confer a significant fitness advantage or disadvantage relative to WT with respect to cell growth on fermentable or nonfermentable carbon sources (Figure S2), growth in the presence of a variety of drugs (Figure S2), sporulation (Figure S3), or growth in a head-to-head competition assay (Figure S4). The one exception occurred when strains were grown on rapamycin, where loss of either *CAF4* or *MDV1* had a negative effect on growth relative to WT, with an additive negative growth defect in the double mutant. It remains to be seen whether this and/or other small differences observed in these studies represent bona fide fitness advantages/disadvantages that are directly attributable to the presence or absence of Caf4.

Finally, we performed domain-swapping experiments in an attempt to identify regions of Caf4 and Mdv1 that might contribute to their functional differences (Figure S5). When expressed from the *MET25* promoter on a plasmid, Mdv1 proteins containing the NTE, CC or WD40 repeats/β-propeller domains from Caf4 rescued mitochondrial morphology defects in a *caf4*Δ *mdv1*Δ strain as well as the WT Mdv1 protein. By contrast, swapping individual Mdv1 domains into the Caf4 protein sequence reduced fission function relative to WT Caf4. Thus, the Caf4 domains have retained fission functions that can substitute for those of Mdv1, however, there is no single domain of Mdv1 that is able to maintain or improve Caf4 function. The inability of Caf4 chimeras containing Mdv1 domains to function as well as WT Caf4 could be due to choice of domain boundaries, altered stability of the chimeric proteins and/or changes in protein structure or binding interfaces critical for fission function.

Our phylogenetic analysis revealed a slightly accelerated rate of amino acid substitution for Caf4 relative to Mdv1. However, both adaptors have retained roles in mitochondrial fission, arguing against the idea that the *CAF4* gene is likely to be lost, like many other paralogs originating from whole gene duplication. Instead, Caf4 and Mdv1 appear to work synergistically, with Mdv1 carrying out the majority of fission in proportion to its higher level of expression. Consistent with this model, we find that Caf4 and Mdv1 are cross-regulated, with Caf4 expression increasing when Mdv1 is absent. The fact that Caf4 has acquired the ability to orient Dnm1 at the cell cortex [18] also supports the idea that the *CAF4* gene has been retained because it confers some type of selective advantage. It remains to be determined whether/how the Dnm1-orienting ability of Caf4 measurably contributes to overall fitness in yeast, and how species like *S. kudriavzevii* have compensated for the loss of Mdv1.

## Materials and Methods

### Growth conditions, strains and plasmids

Yeast strains and plasmids used in this study are listed in supplementary material Table S1 and S2. Unless noted in the Figure legend, standard rich or synthetic dropout media were used for growth, transformation and genetic manipulation of *S. cerevisiae* [27] and *E. coli* [28]. Standard synthetic dextrose medium contains 0.6 mM methionine and cysteine.

The *CAF4* gene encodes two potential initiation codons that could result in methionine at positions 1 and 17 of the predicted protein. To determine whether this 16 amino acid difference at the N-terminus affected Caf4 function, we expressed proteins initiated from each methionine codon under control of the *MET25* promoter. Because we observed no difference in steady-state abundance or fission function of the two proteins (data not shown), the longer version was used for all constructs described in this study.

Plasmids pRS414-*GPD*-*mt-ffRFP* and pRS416-*MET25*-*MDV1* were described previously [21], [29]. pRS414-*GPD*-*mt-mCherry* was generated by PCR amplification of the mCherry open reading frame that was cloned into the EcoRI and XhoI sites of pRS414-*GPD*-mt after digestion and removal of *ffRFP* by the same enzymes. pRS416-*MET25*-*CAF4* was generated by PCR amplification of the *CAF4* open reading frame that was cloned into the BamHI and SalI sites of pRS416-*MET25*. To construct pRS415-*MET25*-*GFP*-*CAF4*, the PCR amplified *CAF4* open reading frame was cloned into the BamHI and SalI sites of pRS416-*MET25*-*GFP*. To construct pRS415-*MET25*-*GFP*-*MDV1*, the PCR amplified *MDV1* open reading frame was cloned into the BamHI and SalI sites of pRS416-*MET15*-*GFP*. To construct pRS416-*MET25*-*ffRFP*-*MDV1*, the PCR amplified *ffRFP* coding region was cloned into the SpeI and BamHI sites of pRS416-*MET25*-*MDV1*. To construct pRS416-*MET25*-*RFP*-*CAF4*, PCR amplified *CAF4* was cloned into the BamHI and SalI sites of pRS-*MET25*-*RFP*.

### Western blotting and protein quantification

Protein expression and abundance was analyzed in yeast whole cell extracts prepared by the alkaline extraction method [30]. 0.25 OD_600_ equivalents of extract was separated by SDS-PAGE and analyzed by Western blotting using the following primary antibodies: anti-yeast actin (1∶5000, J. Cooper, Washington University Saint Louis), α-3-PGK (1∶5000, Invitrogen), anti-Mdv1 (1∶1000, J. Nunnari, U. C. Davis) and anti-Caf4 (1∶250, generated against bacterially expressed, 6His-Caf4 amino acids 175–659). Primary antibodies were detected using fluorescent secondary anti-goat, anti-rabbit or anti-mouse IRDye 800 (LiCor). Fluorescent signals were quantified using an Odyssey scanner and Odyssey 3.0 analysis software (LiCor). In Figure 2B, anti-Caf4 or anti-Mdv1 signals were first normalized to anti-actin signals. The abundance of each protein was subsequently normalized to its abundance in 2.0 mM methionine. In Figure 3B, anti-Caf4 or anti-Mdv1 signals were normalized to anti-3-PGK. In all experiments, data are represented as the mean and SD of three independent experiments.

### Quantification of mitochondrial morphology

Mitochondrial morphology was scored in WT and mutant cells expressing fast folding matrix-targeted red fluorescent protein (mt-ffRFP). Strains were grown at 30°C in selective dextrose synthetic medium and scored in log phase (0.2–0.8 OD_600_). Basal expression levels of Mdv1 and Caf4 proteins from the *MET25* promoter under these conditions result in an approximately five fold increase over endogenous Mdv1 or Caf4 protein levels with no adverse affects on mitochondrial morphology or fission [29](data not shown). For the methionine titration experiments in Figure 2A, *caf4*Δ *mdv1*Δ (adaptor null JSY8612 cells) expressing the indicated Caf4 and Mdv1 proteins from the *MET25* promoter were grown in selective dextrose medium at 30°C overnight. Cultures were diluted to 0.2 OD_600_ in selective dextrose medium lacking cysteine and containing the indicated concentrations of methionine to suppress expression from the *MET25* promoter. Cultures were grown for 3 hours prior to scoring. A Zeiss Axioplan 2 microscope equipped with a 100x oil immersion objective was used to visualize and quantify mitochondrial morphology. Mitochondrial phenotypes were scored in 100 cells, and data are represented as the average and SD of three independent experiments.

### Colocalization studies

Log phase *caf4*Δ *mdv1*Δ cells expressing GFP- or RFP-labeled Mdv1 and Caf4 were grown in standard selective dextrose medium, fixed in 4% formaldehyde (Fisher Scientific) for five minutes at room temperature and washed three times with phosphate buffered saline (PBS, 137 mM NaCl, 2.7 mM KCl, 4.3 mM Na_2_HPO_4_, 1.47 mM KH_2_PO_4_, pH 7.4). Cells were imaged on a Nikon fluorescence microscope using a 100X oil immersion objective, EGFP and DsRed filters and a Coolsnap HQ digital camera (Phototmetrics). Z-stacks (0.2 µm optical sections) were acquired and processed using the DeltaVisionRT system and accompanying DeltaVision software (Applied Precision, Issaquah, WA). Colocalization of GFP and RFP signals was determined in three dimensions using the Orthogonal View function of the DeltaVision software. The number of GFP puncta colocalized with RFP puncta was quantified and normalized to the total number of RFP puncta in each cell (n = 10 cells). Bars and error bars represent the average and SD of three independent experiments.

### Time-lapse imaging

For dual-color time-lapse imaging, log phase *caf4Δ mdv1Δ* cells expressing GFP-Caf4 and mitochondrial targeted RFP were grown in standard selective synthetic dextrose medium and applied to concanavalin A (2 mg/ml, Sigma) treated Lab-tek II Chamber wells (Thermo Scientific) maintained at 30°C. Z-stacks (0.2 µm optical sections) of fields of cells were acquired every seven seconds over a 20 minute time course using a 3-I Marianas Live Cell Imaging microscope workstation (Denver, CO), equipped with dual ultra-sensitive Cascade II 512B EMCCD cameras (Roper Scientific, RS) configured with a Roper Dual-cam and Sutter DG-4 Illuminator (Sutter Instruments) for simultaneous two-channel TIRF/fluorescence acquisition with a 100X, 1.45 NA Plan-Apochromat objective (Zeiss). Data were deconvolved and analyzed using SlideBook 4.2 software (Intelligent Imaging Innovations, Inc). Substacks containing fission events were isolated from the entire stack to minimize signal background and assembled in Photoshop (CS3, Adobe). Brightness and contrast were adjusted using only linear operations applied to the entire image.

Three-color time-lapse imaging was carried out as described above with the following changes. Log phase *caf4*Δ *mdv1*Δ cells expressing integrated Cerulean-Caf4 and EYFP-Mdv1 from the *MET25* promoter (JSY9774) and plasmid-borne mt-mCherry were grown overnight at 30°C in selective synthetic dextrose medium. Cultures were diluted to 0.3 OD_600_ in medium lacking cysteine and containing 1.0 mM methionine 3 hours before imaging. Z-stacks (0.2 µm optical sections) of fields of cells were acquired every ten seconds over a 25 minute time course using a CerFP/EYFP/mCherry filter set (Semrock) and dual Cascade II 512B EMCCD cameras and Sutter DG-4 illuminator (Sutter Instruments) for imaging EYFP, mCherry, and Cerulean signals.

### Phylogenetic Analysis

Blast (NCBI) was used to identify homologs of Caf4 and Mdv1. The genomic sequences of homologs were identified via the Saccharomyces Genome Database and GOLD (Genomes OnLine Database). Predicted protein sequences used to generate this tree were obtained from: YKR036C (*S. cerevisiae CAF4*), YJL112W (*S. cerevisiae MDV1*), PORF 13363 (*S. paradoxus CAF4*), PORF 11728 (*S. paradoxus MDV1*), PROF 13800 (*S. mikatae CAF4*), Contig 2819.8 (*S. mikatae MDV1*), PROF 15127 (*S. bayanus CAF4*), PROF 12617 (*S. bayanus MDV1*), Contig 1606.5 (*S. kudriavzevii CAF4*), GS115 (*P. pastoris ortholog*), KLTH0E15576p (*L. thermotolerans ortholog*), Af293 (*A. fumigatus ortholog*). Amino acid sequences were aligned using ClustalW2 (European Bioinformatics Institute) and indels were removed. PhyML (European Bioinformatics Institute) was used to calculate and assemble the phylogenetic tree.

## Supporting Information

Figure S1 **Growth and peroxisome phenotypes of strains lacking** ***CAF4, MDV1*** **or both adaptors**. (**A**) Growth curve of indicated strains grown in synthetic dextrose containg 0.1% oleic acid. (**B**) Representative Differential Interference Contrast (DIC) and RPF-SKL peroxisomal images in the indicated strains. Scale bar: 2 μm. (**C**) Percentage of cells containing the indicated number of peroxisomes in the indicated strains (n = 50). Bars and error bars are the mean and SD of three independent experiments.
(TIF)

Figure S2 **Growth of strains lacking** ***CAF4, MDV1*** **or both under different conditions.** Ten fold dilutions of the indicated strains were spotted on synthetic solid medium containing the indicated carbon sources or synthetic dextrose medium containing the indicated chemicals or vehicle (DMSO used to solubilize FCCP and CCCP and ethanol used to solubilize antimycin A, oligomycin and rapamycin ). Strains were grown for three days at 30°C.
(TIF)

Figure S3 **Role of Caf4 and Mdv1 in mitochondrial inheritance during sporulation.** (**A**) Quantification of spores per ascus in the indicated strains. n = 1600 (WT), 1501 (*caf4*Δ *mdv1*Δ) and 1500 (*caf4*Δ or *mdv1*Δ). (**B**) Quantification of mitochondrial inheritance by spores in the indicated strains (n = 100). Bars represent the mean of the three independent experiments.
(TIF)

Figure S4 **Competition between isogenic** ***CAF4*** **and** ***caf4***Δ **strains in liquid culture.** PCR amplification at the *CAF4* locus followed by densitometry was used to calculate the fraction of a strain containing the WT *CAF4* gene or a strain containing a *URA3* disruption at the *CAF4* native locus in a mixed culture grown as described in supplemental Materials and Methods S1 for fifteen days (expressed as the ratio of *CAF4*/*caf4::URA3*). Note that there is little change in the ratio of the two strains over time. Bars and error bars are the mean and SD of three independent experiments.
(TIF)

Figure S5 **Mitochondrial fission function of Caf4/Mdv1 domain chimeras.** The amino acid boundaries of the domains in Caf4 and Mdv1 are shown at the top and were determined using a combination of structural information [20], the MultiCoil CC prediction program [31] and sequence alignments of the Caf4 and Mdv1 amino acid sequences [32]. Bottom, the ability of the indicated chimeric proteins expressed from the *MET25* promoter to rescue mitochondrial morphology defects in the *caf4Δ mdv1Δ* strain was quantified (n = 100). Data are represented as the average and SD of three independent experiments.
(TIF)

Table S1 **Plasmids used in this study.** See supporting information.
(DOCX)

Table S2 **Yeast strains used in this study.** See supporting information.
(DOCX)

Materials and Methods S1 See supporting information.
(DOCX)
