# Supplementary figures and images for: The Mitochondrial Fission Adaptors Caf4 and Mdv1 Are Not Functionally Equivalent
Source: PLoS One. 2012 Dec 31;7(12):e53523. doi: 10.1371/journal.pone.0053523 (PMC3534038; doi:10.1371/journal.pone.0053523)

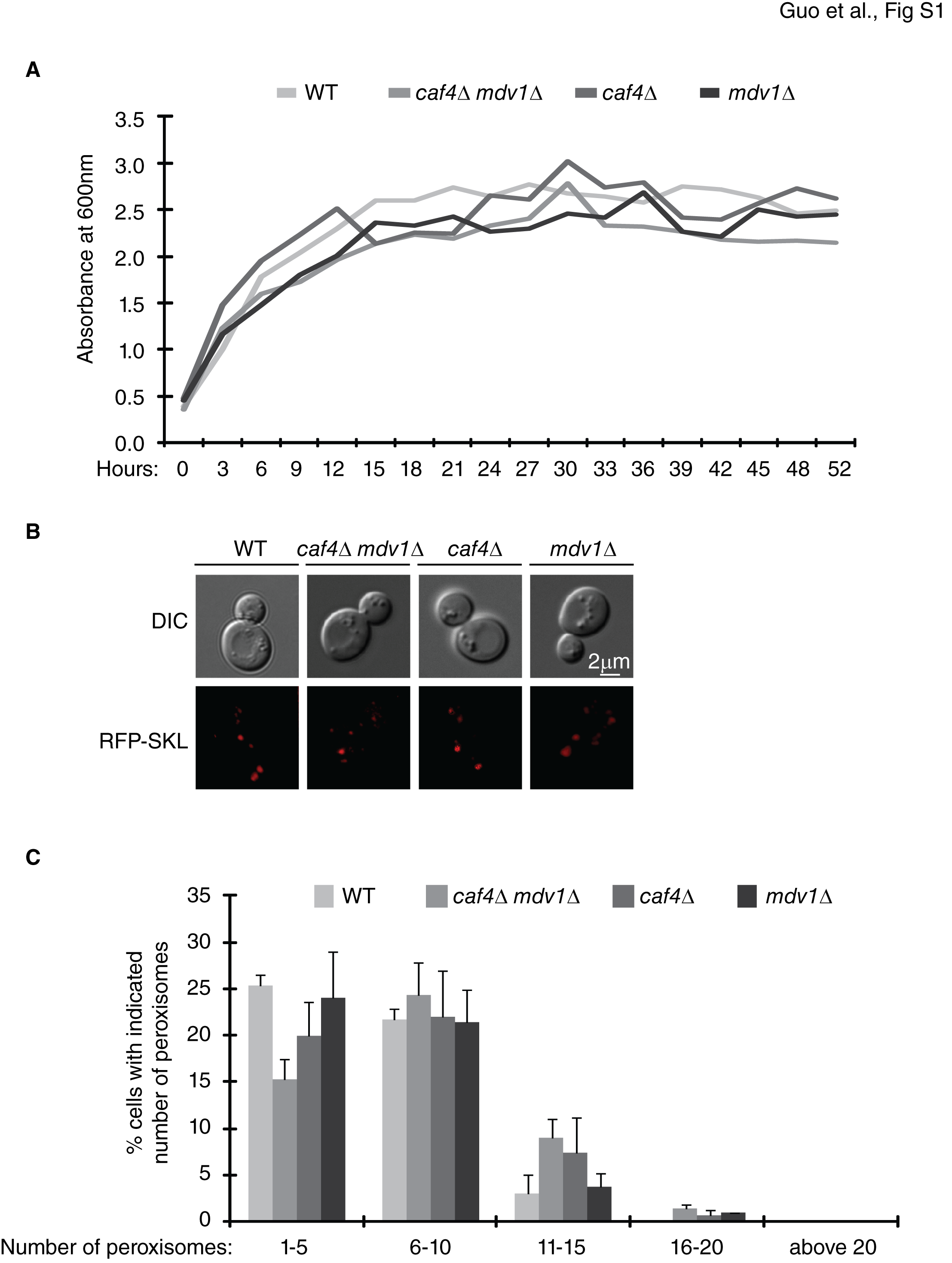

Supplement: Figure S1 — Growth and peroxisome phenotypes of strains lacking CAF4, MDV1 or both adaptors. (A) Growth curve of indicated strains grown in synthetic dextrose containg 0.1% oleic acid. (B) Representative Differential Interference Contrast (DIC) and RPF-SKL peroxisomal images in the indicated strains. Scale bar: 2 μm. (C) Percentage of cells containing the indicated number of peroxisomes in the indicated strains (n = 50). Bars and error bars are the mean and SD of three independent experiments. (TIF) [file pone.0053523.s001.tif]

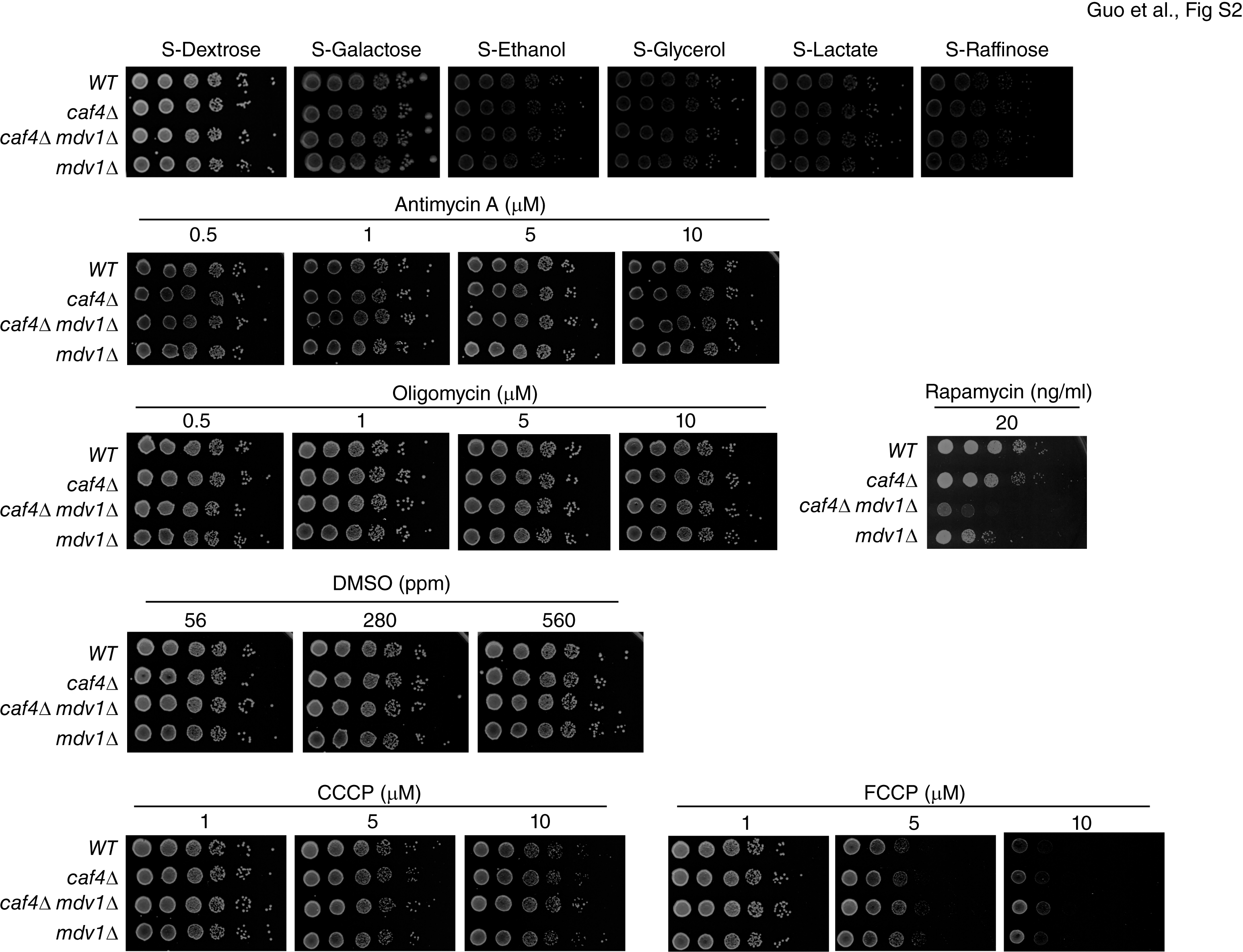

Supplement: Figure S2 — Growth of strains lacking CAF4, MDV1 or both under different conditions. Ten fold dilutions of the indicated strains were spotted on synthetic solid medium containing the indicated carbon sources or synthetic dextrose medium containing the indicated chemicals or vehicle (DMSO used to solubilize FCCP and CCCP and ethanol used to solubilize antimycin A, oligomycin and rapamycin ). Strains were grown for three days at 30°C. (TIF) [file pone.0053523.s002.tif]

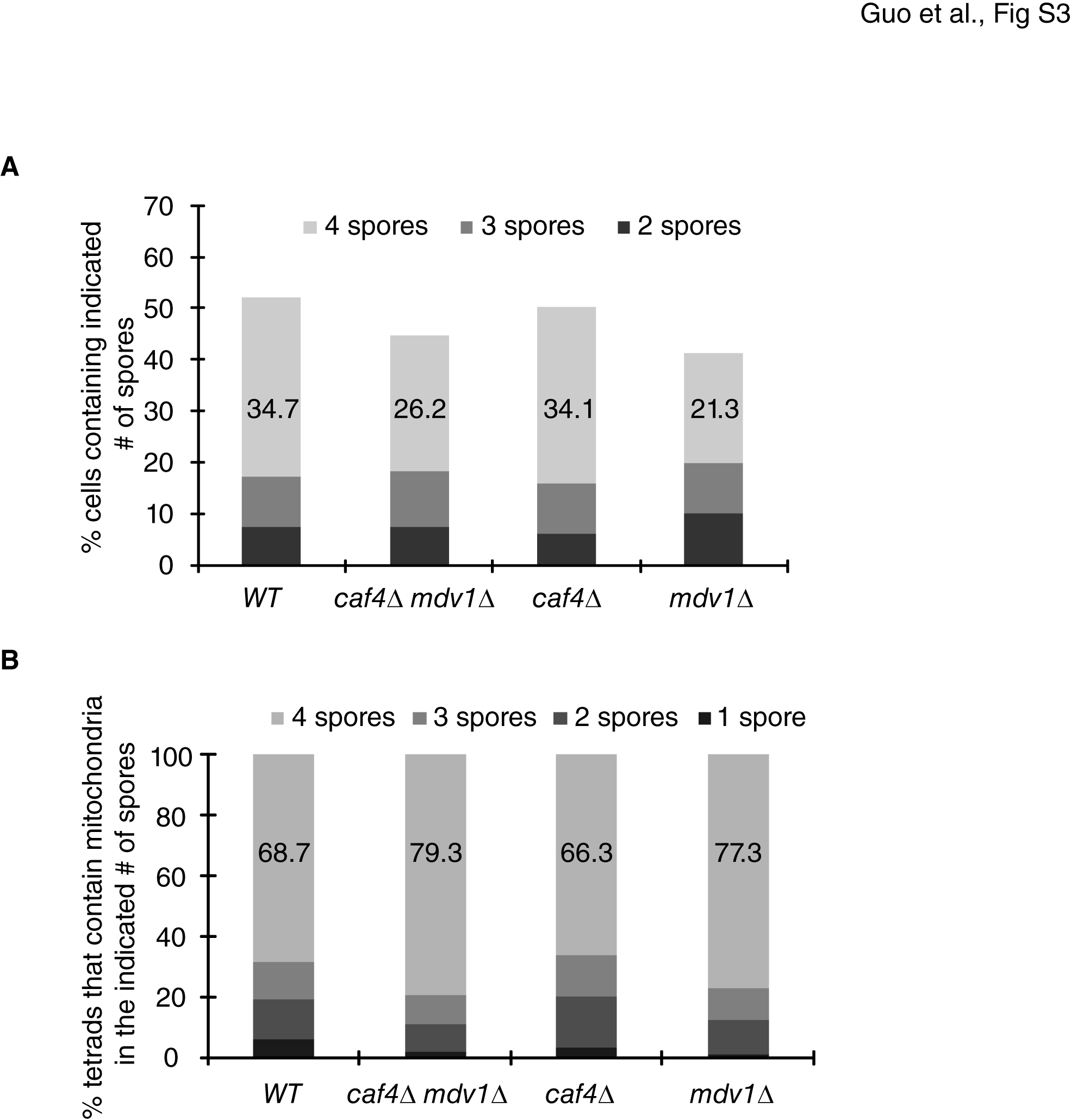

Supplement: Figure S3 — Role of Caf4 and Mdv1 in mitochondrial inheritance during sporulation. (A) Quantification of spores per ascus in the indicated strains. n = 1600 (WT), 1501 (caf4Δ mdv1Δ) and 1500 (caf4Δ or mdv1Δ). (B) Quantification of mitochondrial inheritance by spores in the indicated strains (n = 100). Bars represent the mean of the three independent experiments. (TIF) [file pone.0053523.s003.tif]

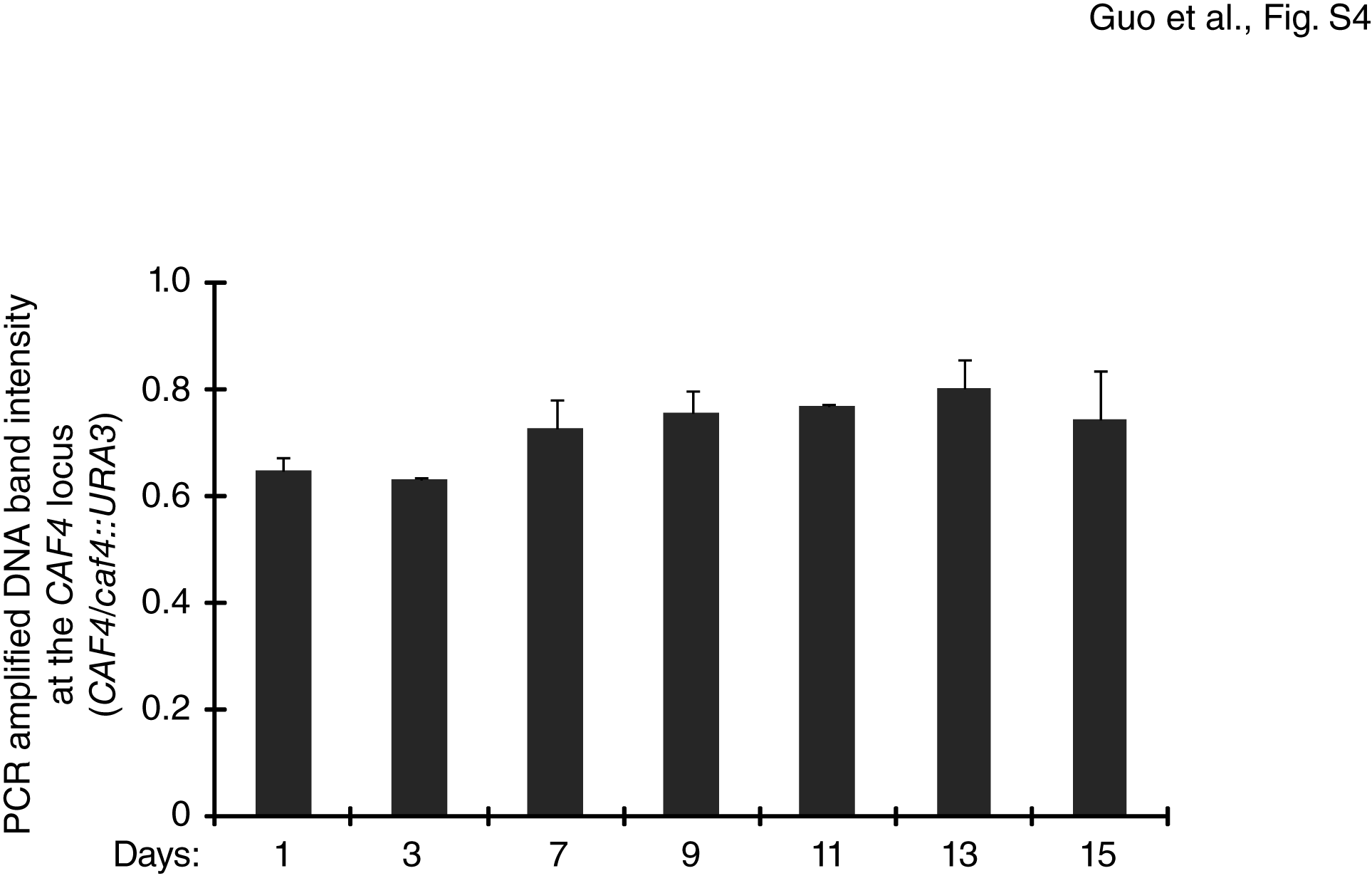

Supplement: Figure S4 — Competition between isogenic CAF4 and caf4Δ strains in liquid culture. PCR amplification at the CAF4 locus followed by densitometry was used to calculate the fraction of a strain containing the WT CAF4 gene or a strain containing a URA3 disruption at the CAF4 native locus in a mixed culture grown as described in supplemental Materials and Methods S1 for fifteen days (expressed as the ratio of CAF4/caf4::URA3). Note that there is little change in the ratio of the two strains over time. Bars and error bars are the mean and SD of three independent experiments. (TIF) [file pone.0053523.s004.tif]

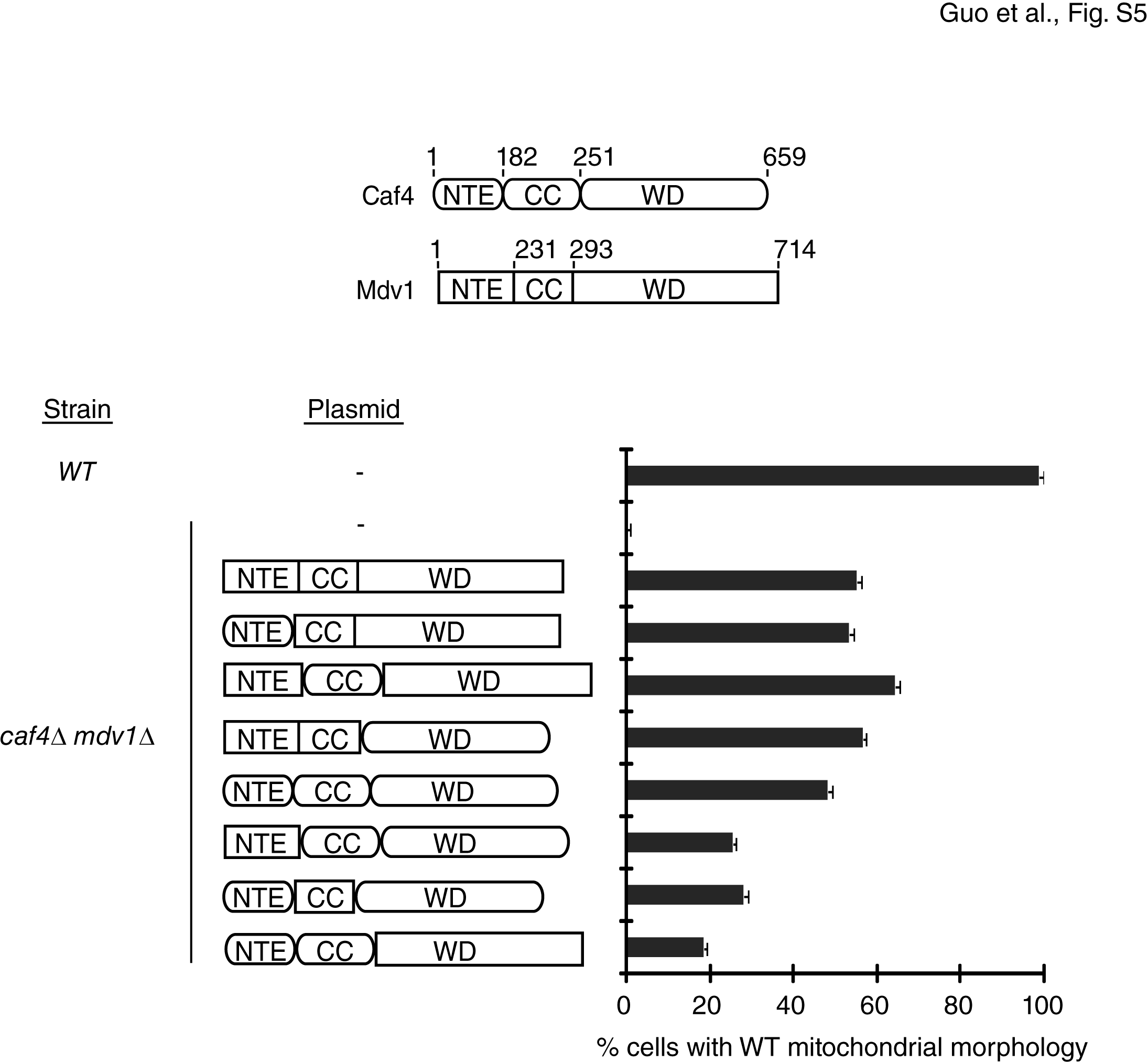

Supplement: Figure S5 — Mitochondrial fission function of Caf4/Mdv1 domain chimeras. The amino acid boundaries of the domains in Caf4 and Mdv1 are shown at the top and were determined using a combination of structural information [20], the MultiCoil CC prediction program [31] and sequence alignments of the Caf4 and Mdv1 amino acid sequences [32]. Bottom, the ability of the indicated chimeric proteins expressed from the MET25 promoter to rescue mitochondrial morphology defects in the caf4Δ mdv1Δ strain was quantified (n = 100). Data are represented as the average and SD of three independent experiments. (TIF) [file pone.0053523.s005.tif]
