# Supplementary material for: The Mitochondrial Fission Adaptors Caf4 and Mdv1 Are Not Functionally Equivalent
Source: PLoS One. 2012 Dec 31;7(12):e53523. doi: 10.1371/journal.pone.0053523 (PMC3534038; doi:10.1371/journal.pone.0053523)
Supplement: Table S1 — Plasmids used in this study. See supporting information. (DOCX) [file pone.0053523.s006.docx]

**Table S1. Plasmids used in this study**

| ID number | Plasmid | Protein Expressed | Reference |
| --- | --- | --- | --- |
| B493 | pRS415-*MET25* | None | ATCC 87322 |
| B494 | pRS416-*MET25* | None | ATCC 87324 |
| B1642 | pRS414-*GPD*-*mt-ffRFP* | *N. crassa* ATP9(1-69) + fast folding DsRed (RFP) | Karren et al., 2005 |
| B2053 | pRS416-*MET25*-*MDV1* | Mdv1 | Karren et al., 2005 |
| B2212 | pRS416-*MET25*-*CAF4* | Caf4 | This Study |
| B2290 | pRS415-*MET25-GFP-CAF4* | GFP-Caf4 | This Study |
| B2291 | pRS416-*MET25-ffRFP-MDV1* | RFP-Mdv1 | This Study |
| B2384 | pRS415-*MET25-GFP-MDV1* | GFP-Mdv1 | This Study |
| B2408 | pRS416-*MET25-ffRFP-CAF4* | RFP-Caf4 | This Study |
| B3002 | p414-*GPD-mt-mCherry* | *N. crassa* ATP9(1-69) + mCherry | This Study |
| B2562 | pRS316-*RFP-SKL* | RFP-SKL | [Fagarasanu](http://jcb.rupress.org/search?author1=Andrei+Fagarasanu&sortspec=date&submit=Submit) et al., 2009 [1] |
| B3174 | pRS416-*MET25*-*CAF4^NTE^-MDV1^CC+WD^* | Caf4^NTE^-Mdv1^CC+WD^ | This Study |
| B3175 | pRS416-*MET25*-*MDV1^NTE^-CAF4^CC^-MDV1^NTE^* | Mdv1^NTE^-Caf4^CC^-Mdv1^NTE^ | This Study |
| B3176 | pRS416-*MET25*-*MDV1^NTE+CC^-CAF4^WD^* | Mdv1^NTE+CC^-Caf4^WD^ | This Study |
| B3177 | pRS416-*MET25*-*MDV1^NTE^-CAF4^CC+WD^* | Mdv1^NTE^-Caf4^CC+WD^ | This Study |
| B3178 | pRS416-*MET25*-*CAF4^NTE^-MDV1^CC^-CAF4^NTE^* | Caf4^NTE^-Mdv1^CC^-Caf4^NTE^ | This Study |
| B3179 | pRS416-*MET25*-*CAF4^NTE+CC^-MDV1^WD^* | Caf4^NTE+CC^-Mdv1^WD^ | This Study |

**Supporting Reference**

1. Fagarasanu A, Mast FD, Knoblach B, Jin Y, Brunner MJ, et al. (2009) Myosin-driven peroxisome partitioning in *S. cerevisiae*. J Cell Biol 186: 541-554.
