# Supplementary material for: The Mitochondrial Fission Adaptors Caf4 and Mdv1 Are Not Functionally Equivalent
Source: PLoS One. 2012 Dec 31;7(12):e53523. doi: 10.1371/journal.pone.0053523 (PMC3534038; doi:10.1371/journal.pone.0053523)
Supplement: Table S2 — Yeast strains used in this study. See supporting information. (DOCX) [file pone.0053523.s007.docx]

**Table S2. Yeast strains used in this study**

| Strain ID | Mating type | Genotype | Reference |
| --- | --- | --- | --- |
| JSY5740 | *MAT****a*** | *ura3-52 leu2∆1 his3∆200 trp1∆63* | Koirala et al., 2010 |
| JSY5750 | *MATalpha* | *ura3-52, leu2∆1, his3∆200, trp1∆63* | This Study |
| JSY8612 | *MAT****a*** | *ura3-52 leu2∆1, his3∆200, trp1∆63, caf4::KanMx, mdv1::HIS3* | Koirala et al., 2010 |
| JSY8613 | *MATalpha* | *ura3-52, leu2∆1, his3∆200, trp1∆63, mdv1::HIS3* | This Study |
| JSY8614 | *MAT****a*** | *ura3-52 leu2∆1 his3∆200 trp1∆63 caf4::KanMx* | This Study |
| JSY8615 | *MATalpha* | *ura3-52, leu2∆1, his3∆200, trp1∆63, lys2∆202, caf4::KanMx* | This Study |
| JSY8616 | *MAT****a*** | *ura3-52 leu2∆1 his3∆200 trp1∆63 mdv1::HIS3* | This Study |
| JSY8618 | *MATalpha* | *ura3-52, leu2∆1, his3∆200, trp1∆63, caf4::KanMx, mdv1::HIS3* | This Study |
| JSY9774 | *MAT****a*** | *ura3-52 leu2∆1 his3∆200 trp1∆63 lys2∆202 caf4::KanMX mdv1::MET-EYFP-MDV1 ho::MET25-Cerulean-CAF4* | This Study |
| JSY9886 | *MAT****a*** | *ura3-52 leu2∆1 his3∆200 trp1∆63 lys2∆202 caf4::KanMX mdv1::CAF4* | This Study |
| JSY9870 | *MATalpha* | *ura3-52, leu2∆1, his3∆200, trp1∆63, lys2∆200, caf4::URA3* | This Study |
| JSY9903 | *MAT****a*** | *ura3-52 leu2∆1 his3∆200 trp1∆63 caf4::MDV1, mdv1::HIS3* | This Study |
| JSY9938 | *MATalpha* | *URA3, leu2∆1, his3∆200, trp1∆63, lys2∆200, CAF4* | This Study |
