# Supplementary material for: The Mitochondrial Fission Adaptors Caf4 and Mdv1 Are Not Functionally Equivalent
Source: PLoS One. 2012 Dec 31;7(12):e53523. doi: 10.1371/journal.pone.0053523 (PMC3534038; doi:10.1371/journal.pone.0053523)
Supplement: Materials and Methods S1 — See supporting information. (DOCX) [file pone.0053523.s008.docx]

**Supplemental Materials and Methods**

**Strains and plasmids**

Yeast strains and plasmids used in supplemental studies are included in Tables S1 and S2. Unless noted, standard rich or synthetic dropout media were used for growth, transformation and genetic manipulation of *S. cerevisiae* [27] and *E. coli* [28].

Plasmid pRS414-*GPD*-*mt-ffRFP* [21,29] was used to visualize and score mitochondrial morphology in the chimera and sporulation studies. pRS416-*MET25*-*MDV1^NTE^-CAF4^CC+WD^* and pRS416-*MET25*-*CAF4^NTE+CC^-MDV1^WD^* were constructed as follows. *CAF4* was PCR amplified with primers containing terminal BamHI and EagI sites and cloned between the same sites in pRS416-*MET25* to generate pRS416-*MET25-CAF4*. pRS416-*MET25-CAF4* was digested with EcoRV and XbaI (to produce a gap within the *CAF4^NTE^*) or SalI and XbaI (to produce a gap within the *CAF4^WD^*). Fragments containing *MDV1^NTE (1-690NT)^* and *MDV1^WD(877-2145NT)^* were PCR amplified and the DNA fragments were co-transformed with EcoRV and XbaI digested pRS416-*MET25-CAF4* or XhoI and SalI digested pRS416-*MET25-CAF4* to generate pRS416-*MET25*-*MDV1^NTE^-CAF4^CC+WD^* and pRS416-*MET25*-*CAF4^NTE+CC^-MDV1^WD^*, respectively, by gap repair. pRS416-*MET25*-*CAF4^NTE^-MDV1^CC^-CAF4^NTE^* was constructed by altering the *CAF4* sequence T^754^TGGAA^759^ in pRS416-*MET25-CAF4* to C^754^TCGAG^759^ by site-directed mutagenesis. This mutagenesis introduced an XhoI site but did not alter the amino acid sequence of the encoded protein. The resulting construct was digested with XhoI and XbaI to remove *CAF4^CC(517-759NT)^*, and PCR amplified *MDV1^CC(691-876NT)^* was cloned between these sites. To construct pRS416-*MET25*-*CAF4^NTE^-MDV1^CC+WD^* and pRS416-*MET25*-*MDV1^NTE+CC^-CAF4^WD^*, *MDV1* was PCR amplified with primers containing terminal BamHI and SalI sites and cloned between the same sites of pRS416-*MET25*. pRS416-*MET25-MDV1* was digested with BamHI and XmaI (to generate a gap within the *MDV1^NTE^*) or XhoI and SalI (to generate a gap within the MDV1*^WD^*) and cotransformed with PCR amplified *CAF4^NTE (1-516NT)^* and *CAF4^WD40 (760-1980NT)^* into JSY8612 to generate pRS416-*MET25*-*CAF4^NTE^-MDV1^CC+WD^* and pRS416-*MET25*-*MDV1^NTE+CC^-CAF4^WD^*, respectively, by gap repair. pRS416-*MET25*-*MDV1^NTE^-CAF4^CC^-MDV1^NTE^* was constructed by digesting pRS416-*MET25-MDV1* with XmaI and XhoI to remove *MDV1^CC(691-876NT)^*. *CAF4^CC(517-759NT)^* was then PCR amplified and cloned into the XmaI and XhoI sites.

**Quantification of mitochondrial morphology**

Mitochondrial morphology was scored in WT and chimera-expressing yeast cells using the pRS414-*GPD*-*mt-ffRFP* plasmid as described in the materials and methods for the accompanying paper.

**Quantification of peroxisome morphology**

Peroxisome morphology was scored in the indicated strains expressing peroxisome-targeted red fluorescent protein (RFP-SKL) [33]. Strains were grown at 30^°^C in selective dextrose synthetic medium, pelleted, washed and diluted in oleic acid containing SD medium (0.67% YNB, 1.97% (NH_4_)_2_SO_4_, 0.1% Glucose, 0.1% Yeast extract, 0.05% Tween 40, 0.1% Oleic acid, pH 6.0). The cultures were grown at 30^°^C with agitation. For growth curves, cell densities were measured every three hours for 52 hours.

Peroxisome number per cell was quantified in WT and mutant strains three hours after dilution in oleic acid containing SD medium. Cells were visualized on an Axioplan 2 microscope (Carl Zeiss, Inc.) equipped with a 100× NA 1.4 oil immersion objective. Peroxisome number per cell was scored in 50 cells, and data are represented as the average and SD of three independent experiments. Representative fluorescence and DIC images were acquired using a monochrome digital camera (AxioCam MRm; Carl Zeiss, Inc.). Z stacks of 0.2-µm slices were deconvolved using AxioVision software (version 4.6; Carl Zeiss, Inc.). Three-dimensional projections of peroxisomes were generated with the transparency (voxel) setting and converted to a single image. Final images were processed and assembled using Photoshop and Illustrator (CS3; Adobe). Brightness and contrast were adjusted using only linear operations applied to the entire image.

**Quantification of sporulation and mitochondrial distribution in spores**

Overnight cultures of WT and fission mutant diploid strains, JSY5740 x JSY5750 (WT/WT), JSY8612 x JSY8618 (*caf4∆ mdv1∆*/*caf4∆ mdv1∆*), JSY8614 x JSY8615 (*caf4∆ MDV1*/*caf4∆ MDV1*) and JSY8616 x JSY8617 (*CAF4 mdv1∆*/*CAF4 mdv1∆*), expressing fast-folding matrix-targeted red fluorescent protein (mt-ffRFP) were harvested, washed three times and diluted to ~0.2 OD_600_ in sporulation medium (20% Raffinose, 1% KAc, 10µm of Adenine, arginine, histidine, isolucine, leucine, lysine, methionine, phenylalanine, threonine, uracil, and valine). The cultures were incubated at 25^o^C for 14 days with agitation after which sporulation efficiency and mitochondrial distribution were scored using DIC and fluorescent imaging, respectively.

**Yeast competition experiments**

For the head-to-head competition assays, overnight cultures of JSY9824 and JSY8667 were mixed (final concentration 0.02 OD_600_ units of each strain) in SD medium and grow at 30^o^C with agitation. 4.0 OD_600_ cell equivalents from the mixed culture were harvested every two days followed by dilution of the culture to 0.02 OD_600_ in fresh medium. The genomic samples were extracted from cell pellets using a MasterPure^TM^ yeast DNA purification kit (Epicentre Biotechnologies). Genes at the *CAF4* locus from both strains were PCR amplified and separated by DNA agarose gel electrophoresis. The abundance of DNA fragments specific for each strain at this locus were imaged and analyzed using a ChemiDoc™ MP System and Quantity One V4.6.6 software (Bio-Rad). The data are represented as the average and SD of three independent experiments.

**Growth Assays**

For serial dilutions, strains JSY5740, JSY8612, JSY8614 and JSY8616 were grown in SD medium to early log phase (OD_600_ 0.5–1.0), pelleted, and resuspended at 0.5 OD_600_. Aliquots of 1:10 serial dilutions were spotted onto synthetic media with the indicated carbon sources (2% Dextrose, 2% Galactose, 3% Ethanol, 2% Glycerol, 2% Sodium lactate or 2% Raffinose) or SD plates containing one of the following chemicals, DMSO (56, 280 and 560 ppm, Mallinckrodt), Antimycin A (0.5, 1, 5, 10µM, Sigma Aldrich), CCCP (1, 5, 10µM, Sigma Aldrich), FCCP (1, 5, 10µM, Sigma Aldrich), Oligomycin (0.5, 1, 5, 10µM, EMD Millipore) and Rapamycin (20ng/ml, EMD Millipore). The strains were grown for 3 days at 30^o^C.
